# Payment perception in the emergency department: The mediating role of perceived quality of healthcare and patient satisfaction

**DOI:** 10.1097/MD.0000000000038527

**Published:** 2024-06-07

**Authors:** Alina Abidova, Pedro Alcântara da Silva, Sérgio Moreira

**Affiliations:** a NOVA University of Lisbon, National School of Public Health, Lisbon, Portugal; b University of Lisbon, Institute of Social Sciences, Lisbon, Portugal; c University of Lisbon, Faculty of Psychology, Lisbon, Portugal.

**Keywords:** doctors, emergency department, meeting expectations, patient satisfaction, payment perception, perceived quality of healthcare, privacy

## Abstract

The aim of this research is to identify the main factors associated with patients’ payment perception and the effects of these factors on payment perception. Patients admitted between January and December 2016 at an emergency department of a public hospital in Lisbon, Portugal, were included in this study, with a representative sample size of 382 patients. A 5% margin of error and a 95% confidence interval were used, and all the data were collected between May and November 2017. To test the mediation models, stepwise multiple linear regression analysis was used. The effect of doctors on payment perception through satisfaction and through perceived quality of healthcare (PQHC) is explained by 3% and 4% of the variation, respectively, with statistically significant results (*P* < .01). Moreover, the effect of privacy and meeting expectations on payment perception through PQHC is explained by 4% and 4% of the variation, with statistically significant results (*P* < .01). Doctors play a crucial role in understanding the patients’ payment perception (with direct and indirect effects). Mediators, in turn, strengthen this effect, in which the contribution of PQHC is more significant than that of satisfaction.

## 1. Introduction

Patient satisfaction can be either a driver of or a barrier to the professional and financial success of a healthcare provider.^[1]^ Empirical evidence suggests a reciprocal relationship between patient satisfaction and the financial performance of a healthcare institution or facility.^[1]^

The government’s role in healthcare spending is also very important for patient satisfaction.^[2]^ There is a positive association between patient satisfaction and patient age, the number of physicians and nurses, and public health expenditures, while there is negative evidence for the number of hospital beds and private health spending.^[2]^

It is important to note that public health spending is 3 times higher than private spending.^[2]^ If public health spending increases, the probability of patient satisfaction increases dramatically by approximately 3500 times.^[2]^

If a patient is from a high-income country, they are approximately 3400 times more likely to be satisfied with the country’s health system compared to a patient from a low-income country.^[2]^ This stark difference between “low-income” and “high-income” countries reflects the different perceptions that exist among patients from different economic conditions.^[2]^ Patients living in wealthier countries are generally more satisfied with the healthcare system than those living in less wealthy countries.^[2]^ Thus, it appears that wealthier countries are able to maintain higher patient satisfaction rates than less wealthy ones.^[2]^

Understanding emergency department (ED) patient satisfaction is especially important in Portugal, which has the highest number of ED visits per capita among European countries.^[3]^ In Portugal, emergency services are provided through the Integrated System of Medical Emergency (SIEM), which is under the supervision of the Ministry of Health. Services are coordinated by regional urgent patients’ guidance centers (CODU), which also operate at sea.^[4]^ Despite the fragmentation of the Portuguese healthcare system, access to the Portuguese National Health Service is universal, according to the Beveridge model, and emergency services are available and accessible to the population regardless of location or socioeconomic status. In addition, special attention should be paid to health centers, as they play an important role in the Portuguese healthcare system. There are many such healthcare facilities throughout the country. Citizens register for public healthcare at the centers, and most doctors are also based in these facilities. Health centers typically provide doctor/physician services, dental services, maternal and childcare services, and even non-life-threatening emergency care.

Researchers have argued that much remains to be done to make EDs in Portugal more effective.^[5]^ They noted that there is a trend in some European countries to use a dedicated staffing model in EDs. Under this model, EDs are staffed by a team of experienced, competent physicians who work full-time. By contrast, many EDs in Portugal use the classic model, which involves hiring inexperienced, young doctors from different departments who take turns in 12-hour shifts. Researchers have shown that hiring a dedicated team of physicians can help reduce costs and increase productivity, thus maintaining a high quality of care.^[5]^

The payment system has a significant impact on the distribution of medical resources, the overall growth of medical expenses, and medical efficiency and quality.^[6]^ Various studies have shown empirical support for the relationship between quality of care and financial performance.^[7–9]^ Several researchers have found mixed evidence regarding the association between quality and healthcare costs.^[10]^ In total, 34% of the studies included in the review revealed a positive association (a higher quality associated with a higher cost), whereas 30% revealed a negative association, and 36% were imprecise, mixed, or did not report any difference.^[10]^

EDs are considered the most challenging part of the healthcare system.^[11]^ EDs worldwide have varying policies with regard to paying for emergency services. It has been widely established that healthcare payment systems do not promote cost-conscious care.^[12]^ When a payment is made, the patient may only be partly covered by insurance or may even have no insurance at all. Considering the economic pressure that paying hospital bills is likely to place on patients, medical price transparency is an important metric.^[13]^ This involves allowing the patients to participate in decisions regarding the costs and benefits of treatment and allowing the emergency physicians to consider the cost of care.^[13]^ Most emergency doctors believe that they should take cost into account, but they are generally unaware of the prices of the different services.^[14]^ One study showed that hospitals can avoid high patient costs by reducing the length of stay in the ED.^[15]^ Researchers found that an increased length of stay of admitted patients can result in a significant loss of hospital revenue and functional treatment capacity in the ED.^[16]^

The discrepancy between the growing demand for resources and available funding due to the increase in patient numbers causes delays in EDs, which impacts the quality of care.^[11]^ ED delays occur for various reasons, such as long medical diagnostics and examination periods, high patient inflow, lack of beds, lack of trained medical staff, nurses, and attendants, and overcrowding.^[17]^ Decreasing ED crowding can have significant benefits for patients while reducing healthcare costs and increasing hospital revenue.^[15]^

The dominant payment method in most countries is fee-for-service,^[18]^ which results in rapidly increasing medical expenses.^[6]^ According to health policy analysts, fee-for-service payment creates an incentive for doctors to prescribe more services,^[19]^ including unnecessary ones, to increase their income.^[18]^

Many of the issues associated with safety, quality, and healthcare efficiency are due to flaws in the existing fee-for-service payment mechanism.^[12]^ The main flaw is not the fee-for-service payment system, but rather the way in which prices are determined.^[19]^ Firstly, fee-for-service payments lead to an increase in the total cost of health care services.^[19]^ Secondly, fee-for-service payments distort the relative prices of various types of healthcare services.^[19]^ These distorted prices lead to the inefficient allocation of healthcare services.^[19]^

Competitive and efficient pricing approaches/payment methods can focus on incentives for patients, physicians, or both.^[19]^ A better way to balance patient benefits and physician profits is through mixed payment systems.^[20]^ One theoretical study has shown that mixed payment methods can overcome the disadvantages of pure payment methods.^[20]^ However, empirical evidence is mixed regarding whether mixed payment methods are better than pure payment methods at maintaining healthcare quality and containing costs.^[20]^ Under different payment systems, patients’ health conditions may affect doctors’ behaviors in various ways.^[20]^ Thus, patients’ health conditions should be taken into consideration when designing mixed payment systems.^[20]^

In this study, we sought to understand how patients perceive the notion of payment, what factors they associate with it, and whether perceived quality of healthcare (PQHC) and satisfaction play a role as mediators of this effect.

## 2. Methods

### 2.1. Participants and data collection

To calculate our random probabilistic sample size, we used a list of 55,903 patients who entered the ED at the public hospital in Lisbon, Portugal at least once between January 1 and December 31, 2016. All responders were at least 18 years old, able to answer the questions, residents of Portugal, and Portuguese-speaking. We excluded respondents under 18 years old, who were unable to answer the questions, who resided outside Portugal, or who had psychiatric illnesses. When a chosen individual had more than one ED admission in the year under study, we chose the last admission according to the date of admission. A 5% margin of error and a 95% confidence interval were used. The representative sample size comprised 382 patients. The data were collected between May and November 2017.

A sample distribution by age and gender was calculated using several steps. First, we calculated the distribution of the universe with a total number of 55,903 patients. Second, we calculated an ideal distribution from the random, probabilistic sample selection of 382 individuals. Our gender distribution was ultimately sufficiently close to an ideal distribution, with a female prevalence. Our age distribution was harder to control, and here we observed a prevalence of the 31 to 40 group of patients in our case and the 18 to 30 group of patients in the case of ideal distribution, while the 41 to 50 age group and the 80+ age group were sufficiently close to an ideal distribution.

Before sending the questionnaire, we contacted all patients by telephone to obtain their permission to send the questionnaire and consent to participate in the survey. We made telephone calls 3 times during the day at different times of the day. If our attempts to reach a patient were unsuccessful, the patient was classified as not responsive. During the data collection period, we made a total of 4413 telephone calls, just including the first-call attempts and excluding all repeat calls afterwards. Those who did not have a telephone number on our list were excluded prior to the initiation of the calls.

Eventually, 1553 patients agreed to participate and gave permission for the questionnaire to be sent by post office. Only 506 questionnaires were sent due to the study’s financial constraints. We received 143 questionnaires back, and 363 were not returned. With respect to the e-mail distribution, 959 patients agreed to participate and gave permission to send the questionnaire by e-mail. Among them, 340 responded to the questionnaire online, and 619 did not. The total number of obtained questionnaires exceeded the total number of a calculated necessary sample size, resulting in exclusion of 101 incomplete/poorly completed questionnaires where the number of questions answered was very low, as well as questionnaires that were returned after our data analysis had already begun. Thus, among the 382 individuals, 75.9% were online (e-mail) respondents, and 24.1% responded via regular post office.

This study was performed in compliance with the ethical principles set out in the Declaration of Helsinki. Authorization for this study was obtained from the Ethics Committee and Board of Directors and Administration of the Centro Hospitalar de Lisboa Ocidental E.P.E. (CHLO).

### 2.2. Measures and analysis procedure

Our modified-elaborated questionnaire was partly based on the questionnaire used by Pereira et al^[21]^ and was partly based on the Instruments for Evaluating Hospital Quality - Adult Emergency, which was designed, developed, and tested by the Center for Studies and Research in Health of the University of Coimbra.^[22]^ In addition, we took into consideration the fourth national health survey (Portugal) prepared by the National Institute of Health Dr Ricardo Jorge/National Institute of Statistics,^[23]^ as well as the survey used to investigate the aging process in Portugal.^[24]^ The questionnaire was developed using various measurement scales and consisted of 75 questions. It was sent either by post office or e-mail, depending on the respondent’s preference.

With exploratory factor analysis, the variable “doctors” was simplified into a single composite measure. Internal consistency analysis revealed a Cronbach alpha of 0.98 with 88.79% of the explained variance.

We followed a rigorous methodological approach that consisted of an in-depth, step-by-step statistical procedure. Several researchers have demonstrated that quality and satisfaction are distinct concepts, and they have emphasized the importance of satisfaction as a mediator, in contrast to perceived quality.^[25]^ Thus, first of all, in an attempt to understand the differences and/or similarities between satisfaction and PQHC, we decided to run bivariate correlations between all relevant variables. Then, in order to perform a preliminary analysis of the determinants of satisfaction and PQHC, we decided to conduct a multiple regression analysis, including either satisfaction or PQHC as the dependent variables. In this analysis we used 18 predictors (only those with a strong, moderate, or weak correlation with satisfaction and the PQHC). Based on the results obtained in the multiple regression analysis, we chose the variables to include in the mediation models. For the given analysis, we selected only the main predictors (antecedents) of satisfaction/PQHC that we considered as having statistically significant conditions (*P* ≤ .05), and some other predictors that had a statistically significant (marginal effects) relationship with satisfaction/PQHC (*P* ≤ .10) as identified in multiple regression analysis. Thus, regarding satisfaction, we used the following set of variables (predictors): doctors (*r* = 0.14, *P* ≤ .01); qualitative perceived waiting time for triage (*r* = 0.08, *P* ≤ .05); meeting expectations (*r* = 0.53, *P* ≤ .01); and information about possible delays (*r* = 0.06, *P* ≤ .10). Regarding PQHC, we used the following set of variables (predictors): doctors (*r* = 0.43, *P* ≤ .01); meeting expectations (*r* = 0.26, *P* ≤ .01); qualitative perceived waiting time to be called back by the doctor following examinations and/or tests (*r* = 0.10, *P* ≤ .10); privacy (*r* = 0.09, *P* ≤ .10); and accessibility and availability (*r* = 0.09, *P* ≤ .10).

The mediation models were computed with different combinations of the selected variables regarding satisfaction and regarding PQHC. In this research, we considered and analyzed different models with regard to satisfaction and PQHC as they have been proven to be distinct concepts.^[25]^

Thus, variables were selected considering that the antecedents (predictors) were significantly correlated with both the mediators (PQHC or satisfaction) and the dependent variable (agreement with the existence of a fixed user charge payment to access the ED to help improve the services and treatments received, regardless of the current charges and exemptions). Stepwise multiple linear regression analysis was used to test the mediation models using the methodology proposed by Baron and Kenny (1986).^[26]^

## 3. Results

### 3.1. Descriptive statistics

The participants were mostly from Lisbon (96%) and were grouped into persons with dual nationality (2.1%), other nationality (2.6%), and Portuguese (95.3%), with the proportion of females to males at 61.3%:38.7%. The age distribution of participants across age groups was almost uniform: 18 to 30 (14.9%), 31 to 40 (19.1%), 41 to 50 (14.4%); 51 to 60 (17.6%); 61 to 70 (9.2%); 71 to 80 (9.8%); 80+ years (14.7%). The descriptive statistics of the main variables used in the mediation models are represented in Table 1.

**Table 1:** Means, minimum, maximum, and standard deviations.

	n	Mean	Min	Max	SD
Privacy					
The way the privacy was safeguarded	372	7.27	1	10	2.41
Doctors					
Friendliness and helpfulness of the doctor(s)	379	7.74	1	10	2.17
Competence and professionalism of the doctor(s)	374	7.90	1	10	2.15
The way the doctor explained a health problem (diagnosis) during the examination	378	7.78	1	10	2.30
The explanations given by the doctor on the exams performed and the objectives of the treatment to be undertaken	366	7.77	1	10	2.39
The information provided on precautions to be taken, recommendations, and how to take or apply the medications prescribed (written or oral) after leaving hospital	370	7.95	1	10	2.23
Overall, the performance of the doctor(s)	378	7.89	1	10	2.26
Expectations					
Meeting expectations	375	6.65	1	10	2.39
Payment perception					
Agreement with the existence of a fixed user charge payment to access the ED in order to help improve the service and treatments received, regardless of the current charges and exemptions	363	5.88	1	10	3.60
Satisfaction					
Considering the entire experience in the ED, the level of satisfaction	380	7.10	1	10	2.38
Perceived quality of healthcare					
Overall, evaluation of the quality of healthcare	373	7.65	1	10	2.10

ED = emergency department.

### 3.2. Effect on payment perception (with satisfaction)

**Figure 1.**
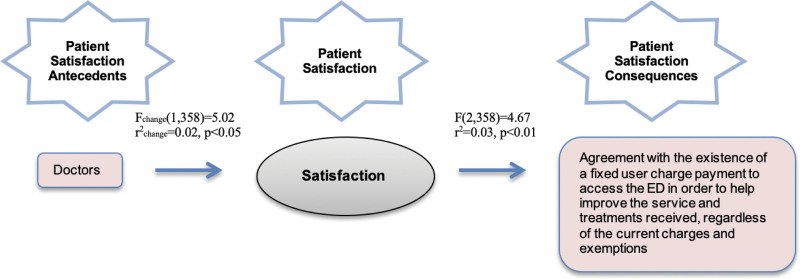
Effect on payment perception (with satisfaction).

Only one mediation model was found to be statistically significant with satisfaction. Thus, only one antecedent (i.e., doctors) can be observed, as represented in Figure 1.

Here, it can be observed that the contribution of satisfaction in the given model is 2% of the explained variance. This explains the effect of doctors on the agreement with the existence of a fixed user charge payment to access the ED to help improve the services and treatments received, regardless of the current charges and exemptions, through satisfaction by 2%, with statistically significant results (*P* < .05).

Without satisfaction as a mediator, the effect of doctors on the agreement with the existence of a fixed user charge payment to access the ED to help improve the services and treatments received, regardless of the current charges and exemptions, is explained by only 1%. Looking at the correlation level, it can be observed that the model without satisfaction as a mediator has an *r* = 0.11 correlation level. Adding satisfaction as a mediator in the model reduces the direct correlation level to *r* = 0.01, showing complete mediation in the model through satisfaction.

Analyzing the entire model shows that 3% of the explained variance is statistically significant (*P* < .01). Thus, it can be concluded that, through satisfaction, this effect is explained by 3% of the variation.

### 3.3. Effect on payment perception (with PQHC)

**Figure 2.**
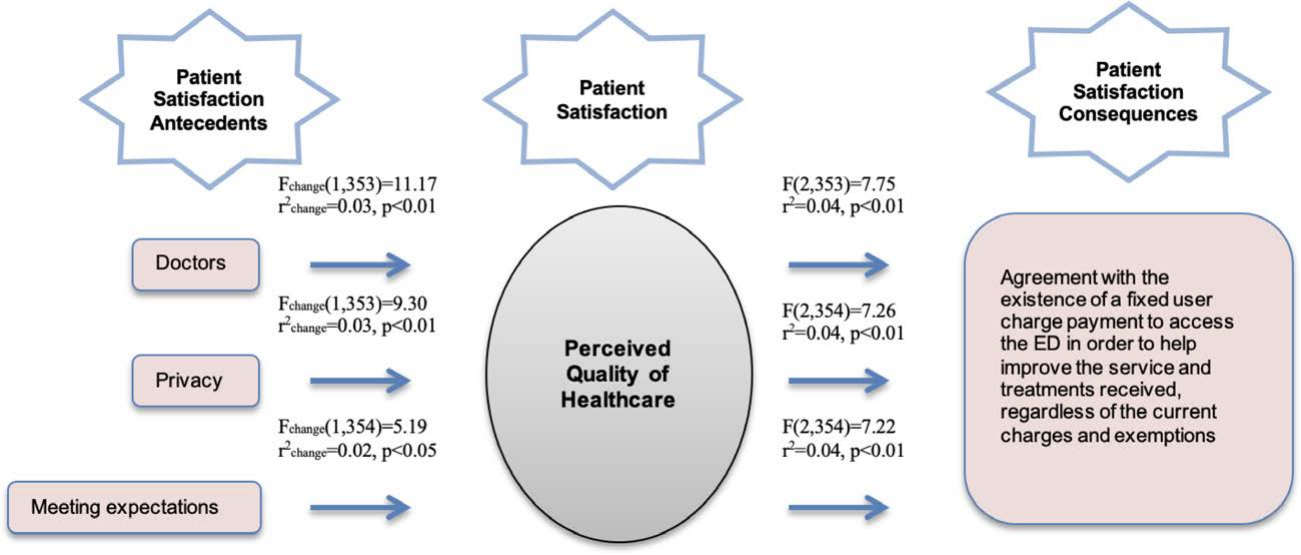
Effect on payment perception (with PQHC). PQHC = perceived quality of healthcare.

Three mediation models were found to be statistically significant with PQHC. Thus, 3 antecedents can be observed (i.e., doctors, privacy, and meeting expectations), as represented in Figure 2.

The first model, which represents doctors, shows that the contribution of PQHC as a mediator in the given model is 3% of the explained variance. This explains the effect of doctors on the agreement with the existence of a fixed user charge payment to access the ED to help improve the services and treatments received, regardless of the current charges and exemptions, through PQHC by 3%, with statistically significant results (*P* < .01).

Without PQHC as a mediator, the effect of doctors on the agreement with the existence of a fixed user charge payment to access the ED to help improve the services and treatments received, regardless of the current charges and exemptions, is explained by only 1%. The correlation level shows that the model without PQHC as a mediator has an *r* = 0.11 correlation level. Adding PQHC as a mediator in the model reduces the direct correlation level to *r* = −0.09, showing partial mediation in the model through PQHC. In this case, the agreement with the existence of a fixed user charge payment to access the ED to help improve the services and treatments received, regardless of the current charges and exemptions, is explained both by a mediation relation and by a direct relation with the predictor.

Analyzing the entire model shows that 4% of the explained variance is statistically significant (*P* < .01). Thus, it can be concluded that, through PQHC, this effect is explained by 4% of the variation.

The second model that represents privacy shows that the contribution of PQHC in the given model is 3% of the explained variance. This explains the effect of privacy on the agreement with the existence of a fixed user charge payment to access the ED to help improve the services and treatments received, regardless of the current charges and exemptions, through PQHC by 3%, with statistically significant results (*P* < .01).

Without PQHC as a mediator, the effect of privacy on the agreement with the existence of a fixed user charge payment to access the ED to help improve the services and treatments received, regardless of the current charges and exemptions, is explained by only 1%. Looking at the correlation level, it can be observed that the model without PQHC as a mediator has an *r* = 0.12 correlation level. Adding PQHC as a mediator in the model reduces the direct correlation level to *r* = 0.04, showing partial mediation in the model through PQHC. In this case, the agreement with the existence of a fixed user charge payment to access the ED to help improve the services and treatments received, regardless of the current charges and exemptions, is explained both by a mediation relation and by a direct relation with the predictor.

For the entire model, 4% of the explained variance is statistically significant (*P* < .01). Thus, it can be concluded that, through PQHC, this effect is explained by 4% of the variation.

The third model that represents meeting expectations shows that the contribution of PQHC in the given model is 2% of the explained variance. This explains the effect of meeting expectations on the agreement with the existence of a fixed user charge payment to access the ED to help improve the services and treatments received, regardless of the current charges and exemptions, through PQHC by 2%, with statistically significant results (*P* < .05).

Without PQHC as a mediator, the effect of meeting expectations on the agreement with the existence of a fixed user charge payment to access the ED to help improve the services and treatments received, regardless of the current charges and exemptions, is also explained by 2%. Looking at the correlation level, it can be observed that the model without PQHC as a mediator has an *r* = 0.16 correlation level. Adding PQHC as a mediator in the model reduces the direct correlation level to *r* = 0.04, showing partial mediation in the model through PQHC. In this case, the agreement with the existence of a fixed user charge payment to access the ED to help improve the services and treatments received, regardless of the current charges and exemptions, is explained both by a mediation relation and by a direct relation with the predictor.

Examining the entire model shows that 4% of the explained variance is statistically significant (*P* < .01). Thus, it can be concluded that, through PQHC, this effect is explained by 4% of the variation.

### 3.4. Payment perception differences

We also decided to examine the differences between those who paid and those who were exempted. The test revealed statistically significant differences between the categories (F(1317) = 8.32, *P* ≤ .01). The participants who paid leaned more towards agreement with the payment of a fixed user charge to access the ED to help improve the services and treatments received, regardless of the current charges and exemptions (M = 6.33, SD = 3.44), than those who were exempted (M = 5.16, SD = 3.68).

## 4. Discussion

Most EDs are not designed to monitor patients longitudinally, making it difficult to track total patient costs.^[27]^ Emergency doctors quite often have only one encounter with a patient, and there is generally a high level of clinical and diagnostic uncertainty.^[12]^ The decisions of physicians have a financial impact on the patients,^[13]^ and the number of patients who are unable to pay their medical bills is growing.^[28]^ Unpaid medical bills are associated with two-thirds of personal bankruptcies.^[13]^ Patients with larger bills and without insurance are at greatest risk of facing a significant healthcare debt burden that they cannot resolve.^[28]^ Most importantly, patients without insurance fail to pay off their debt in full 88% of the time.^[28]^ One study showed that patients with insurance who owed less than $25 and had a high severity case paid 94% of the time, while patients without secondary or primary insurance who owed more than $25 paid only 10% of the time.^[28]^ Our results show that payment may be perceived differently by those who paid and those who were exempted. Namely, the participants who paid leaned more towards agreement with the payment of a fixed user charge to access the ED than those who were exempted.

Physicians are generally unaware of the expenses or charges associated with the treatment they provide, and they significantly underestimate patients’ overall costs and charges (93% of the time).^[29]^ However, ED patients typically do not consider price before going to the ED, and on average, patients overestimate their out-of-pocket cost by $144 and the cost of their care by $2484.^[30]^ One study showed that in all 3 payer groups (uninsured, private, and public), a declining payment rate was primarily the result of rapidly rising charges, whereas payments failed to keep pace with charges.^[31]^

Physician payment models are considered an important strategy for improving quality, access, health, and the value of health care, as payment method has been found to influence physician behavior.^[32]^ Findings from another study indicate that physicians’ involvement in the communication, implementation, design, and ongoing evaluation of pay-for-performance models has an influence on their behavior.^[33]^ Researchers pointed out that fee-for-service physicians tend to see patients more quickly and are prone to overutilization, providing services and prioritizing patients that maximize their income.^[34]^ By contrast, contract-based physicians may have less financial incentive to see patients quickly and may be more focused on quality.^[34]^ Thus, the payment mechanism may or may not change the productivity of an individual physician.^[34]^

Although physician compensation is an important policy issue, it may not be the primary determinant of ED patient throughput or operational efficiency.^[34]^ There can be a complex interplay between practice patterns, preferences for payment models, and physician characteristics.^[35]^ One study found that adding a general practitioner to a hospital-based ED was a cost-effective intervention (less expensive, more effective) compared to a standard ED service in terms of patient satisfaction and process time.^[36]^ Total costs per patient were €288 in the usual care period and €217 in the new care period.^[36]^ Previously, another study also reported that this new method of care had a positive impact on patient satisfaction while maintaining quality of care.^[37]^

Physician well-being and physician satisfaction are not synonymous, but the same factors influence both, which has a notable effect on physician behavior and, thus, the quality of health care they provide.^[38]^ The costs associated with physician burnout are about $7600 per physician per year and $4.6 billion nationally.^[39]^ One study found that emergency physicians had the lowest total payments ($31−$54 per-physician on average annually) in the healthcare industry over a 9-year period (2013–2021).^[40]^ Researchers have noted that the healthcare industry has less impetus to make general payments to emergency physicians because they often provide less expensive pharmacotherapy compared to other specialists and short-term prescriptions.^[41]^

Researchers emphasized that the 3 factors that characterize patients’ primary reasons for seeking ED care (ED preference, convenience, and medical necessity) are themselves correlated.^[42]^ If one were to perceive a medical need, then considerations of immediacy of care, time, location, and quality of care would be factors when choosing the best place for care.^[42]^ What is especially noteworthy is that although these 3 factors are correlated, they are not correlated with financial factors.^[42]^ However, according to our results, patients associate payment mainly with doctors’ friendliness and helpfulness; competence and professionalism; explanations of a health problem (diagnosis); explanations of the exams performed and the objectives of the treatment to be undergone; provision of information on precautions to be taken, recommendations, and how to take or apply the medications prescribed; and overall performance, both directly and indirectly through PQHC and satisfaction. Therefore, it can be concluded that doctors are the ones who primarily contribute to the understanding of payment perception.

Understanding patient expectations can improve patient satisfaction.^[43]^ If expectations are met, this can positively impact patient perceptions and, in turn, improve patient satisfaction ratings.^[44]^ One study highlighted the importance of staff communication in improving the quality of care and meeting patient expectations,^[45]^ while another study showed that up to 70% of litigation involves real or perceived problems with physician communication, which impacts patient expectations.^[43]^ However, healthcare providers in EDs must be aware of another concern related to breaches in confidentiality and privacy.^[46]^ In one study, a total of 36% of patients overheard conversations with similar frequency in curtained and walled rooms.^[46]^ Unprofessional or inappropriate comments by staff were heard by 1.6% of patients.^[46]^ According to our results, privacy and meeting expectations can also have an effect on payment perception directly and indirectly only through PQHC. Thus, satisfaction does not play any role in this case.

Proper reimbursement for emergency physician services is essential for continued availability and access to high-quality care.^[47]^ Efforts to monitor healthcare costs may place more pressure on doctors to justify their decisions.^[13]^ Nevertheless, emergency doctors should not be forced to take significant financial risks or risk for outcomes and costs that are beyond their control.^[12]^ Such a lack of innovation regarding emergency care payment is considered a quite negative finding.^[48]^ Even though little attention has been paid to ED payment, there is an opportunity for policymakers and ED leaders to design value-based payment models to deliver high-quality emergency care.^[48]^ Our results show that there are more factors that influence payment perception through PQHC and with stronger effects than through satisfaction. Thus, PQHC deserves more attention when designing a payment model.

## 5. Limitations

The data collection had some limitations, as it was confined to 1 ED in 1 country. The Portuguese healthcare system is characterized by the Beveridge model. Other countries with different healthcare systems may have different results. In addition, we only considered the Portuguese-speaking population who could answer the questions. In this study, power analysis was not performed prior to the final sample size calculation. We chose a sample distribution with a 5% margin of error and a 95% confidence interval rather than a lower margin of error due to time and financial constraints.

## 6. Conclusion

Mediators (PQHC/satisfaction) play an important role in understanding payment perception. We identified the main predictors of payment perception as doctors, privacy, and meeting expectations. However, mediators play different roles with different predictors. Namely, the contribution of PQHC to understanding payment perception is 3% and 3%, with privacy and doctors as predictors, while with meeting expectations, it is only 2%. Looking at the entire models, with interaction effects, the effect of doctors, privacy, and meeting expectations on payment perception through PQHC is 4%, 4%, and 4%.

We must note that satisfaction plays an important role in understanding payment perception only with doctors as a predictor. Separately, the contribution of satisfaction is 2%, and looking at the entire model, the effect of doctors on payment perception through satisfaction is 3%.

Thus, we can see that PQHC plays a more important role as a mediator than satisfaction in understanding payment perception, while doctors play the most important role as a predictor. However, it is crucial to explore in future research which payment model or method contributes to a better PQHC and higher satisfaction levels among patients and emergency physicians.

## Author contributions

**Conceptualization:** Alina Abidova.

**Data curation:** Alina Abidova.

**Formal analysis:** Alina Abidova, Sérgio Moreira.

**Investigation:** Alina Abidova.

**Methodology:** Alina Abidova, Pedro Alcântara da Silva.

**Project administration:** Alina Abidova.

**Resources:** Alina Abidova.

**Supervision:** Alina Abidova, Pedro Alcântara da Silva.

**Validation:** Alina Abidova.

**Visualization:** Alina Abidova.

**Writing – original draft:** Alina Abidova.

**Writing – review & editing:** Alina Abidova, Pedro Alcântara da Silva, Sérgio Moreira.
